# Prevalence, correlates, and comorbidities of internet gaming disorder and problematic game use: national mental health survey of Korea 2021

**DOI:** 10.3389/fpsyt.2024.1442224

**Published:** 2024-10-15

**Authors:** Young-Mi Ko, Eun Sol Lee, Subin Park

**Affiliations:** ^1^ Division of Mental Health Research, Mental Health Research Institute, National Center for Mental Health, Seoul, Republic of Korea; ^2^ Mental Health Research Institute, National Center for Mental Health, Seoul, Republic of Korea

**Keywords:** internet gaming disorder, problematic game use, comorbidity, prevalence, attention-deficit/hyperactivity disorder

## Abstract

**Background:**

This study investigated the prevalence, correlates, and comorbidities of Internet Gaming Disorder and problematic game use among the general population in Korea.

**Methods:**

The data of 2,764 individuals aged 18 to 49 years who participated in the National Mental Health Survey of Korea 2021 were analyzed. The diagnostic assessments were based on the Structured Clinical Interview for Internet Gaming Disorder and the Composite International Diagnostic Interview. The Game Overuse Screening Questionnaire assessed problematic game use. Multiple logistic regression analyses were performed, and a complex sampling design analysis was applied.

**Results:**

The 12-month prevalence rate of Internet gaming disorder (IGD) was 0.8% and the 1-month prevalence rate of problematic game use was 8.4%. IGD was higher in men, younger people, unemployed, and in those with low physical activity, dissatisfaction with their quality of life, and who perceived more loneliness and social isolation. While both alcohol use disorder (AUD) and Attention-Deficit/Hyperactivity Disorder (ADHD) were significantly associated with IGD, only ADHD was significantly associated with problematic game use.

**Conclusion:**

IGD and problematic game use are relatively prevalent in the Korean adult population and are comorbid with AUD and ADHD. Therefore, a preventive strategy for IGD and problematic game use is needed for game users who are more likely to be addicted, such as younger male users. In addition, mental health screening and appropriate treatment for both game addiction and comorbid psychiatric disorders should be provided to individuals with IGD and problematic game use.

## 1 Introduction

In the current highly digitalized world, gaming represents not only a recreational activity, but also a potential threat when a game user loses with a sense of reality, substituting gaming for social, occupational, or other recreational activities (1). Internet gaming disorder (IGD), the repetitive use of Internet-based games that leads to significant impairment or distress, was deliberated in the Diagnostic and Statistical Manual of Mental Disorders, Fifth Edition (DSM-5) in Section III that was published in 2013, as a condition for further study (2). Gaming disorder was defined as a distinct nosological entity under the addictive disorders section of the 11^th^ Revision of the International Statistical Classification of Diseases and Related Health Problems (ICD-11) (3). However, the concept of IGD is still under debate as clinical data on symptoms of its ‘true’ addiction are lacking, negative consequences may be overemphasized, and Internet gaming could be related to inefficient strategies for dealing with life problems or other underlying mental health problems (4, 5).

Several studies have used instruments based on the DSM-5 to estimate the prevalence rates of problematic game use or game addiction. Rehbein et al. (6) reported a 1.2% prevalence rate in a school-aged population in Germany, and Lemmens et al. (7) found a 5.4% prevalence rate in a Dutch sample of adolescents and young adults. Using a web-based survey, Kim et al. (8) reported that 13.8% of the surveyed Korean adults aged 20–49 years met the DSM-5 criteria for IGD. More recently, Darvesh et al. (9) posited that the prevalence rates of IGD using the DSM-5 criteria varies from 0.21% to 57.5% in the general population. This wide gap between the prevalence rates might originate from differences in sample size, sample characteristics, and age range. For instance, the study using a large sample size of male adults among the general population indicated a low prevalence rate of IGD (10), while a small-scale observational study with online game players who were late teens and young adult males showed a higher prevalence rate (11). The prevalence rates of problematic game use ranged from 0.5 to 9.9%, with the variation attributed to the study population and the diagnostic instrument utilized (12). The prevalence rates of problematic game use in a large general population study varied among adolescents (7.6%), young adults (3.3%), and older adults (3.0%) (13).

Most studies have described a higher prevalence of IGD and problematic game use among young people than among the elderly, who are considered “age at risk” (14–16). Moreover, this prevalence is higher in males than females (14, 17, 18). Several psychological factors, personality traits, and psychological disorders are perceived to increase the risk of IGD (19). Impulsivity, poor self-control, and the pursuit of desired appetitive goals are psychological features associated with IGD (20). Gaming addiction has been reported to be significantly associated with loneliness (21) and low life satisfaction (22). IGD has been associated with loneliness, low self-worth, poor social skills, lack of social acceptance, dysfunctional coping, and psychopathology (23–25). Previous studies have also reported that psychological resilience reduces the effect of individual risk factors on addictive behaviors (26, 27).

A growing body of research suggests that gaming disorders are associated with several other mental health disorders, including depression (28), anxiety (22, 29), problematic substance use (30), and personality disorders (31). Regarding psychiatric comorbidities, some studies have reported no differences in the prevalence of alcohol use disorder (AUD) between problematic game users and usual users; however, illicit drug use is prevalence in problematic game users (32, 33). Of the numerous psychiatric comorbidities of IGD, attention-deficit/hyperactivity disorder (ADHD) is considered the most frequent, due to its prevalence and conceptual overlap (5, 34–36). Yen et al. (36) identified the shared features of impulsivity and hostility in ADHD and IGD.

Because of the limited existing studies that use instruments that are based on the DSM-5 and representative samples, we investigated the prevalence of IGD and problematic game use and examined the correlates and comorbidities of a wide range of psychiatric disorders among a nationwide sample of Korean adults aged 18–49 years.

## 2 Methods

### 2.1 Sample

We examined the data from the National Mental Health Survey of Korea 2021 (NMHSK2021), which was conducted between June 19, 2021 and August 31, 2021. A nationally representative sample of non-institutionalized residents was selected. This study used a complex sample design, in which state(si)/province(do), *dong/eup/myeon*, and household type were used as stratification variables. The NMHSK2021 survey was used to measure the lifetime and 12-month prevalence of major psychiatric disorders and their association with sociodemographic characteristics and comorbidities among Korean adults according to the Diagnostic and Statistical Manual of Mental Disorders, Fourth Edition (DSM-IV). The survey encompassed participants aged between 18 and 79 years during the NMHSK 2021 study period, as per the Population and Housing Census. The interviews involved 13,530 households, with 5,511 participants completing the interviews (37). In this study, 2,764 participants who played internet/online games among people aged 18–49 years were used for the final analysis, following a prior study that indicated an increase in the prevalence of gaming disorder with young age (7, 8, 14, 22, 38–40).

This study was approved by the Institutional Review Board of the National Center for Mental Health (IRB No. 116271-2022-19).

### 2.2 Measurement

#### 2.2.1 Internet gaming disorder

The Structured Clinical Interview for DSM-5 Internet Gaming Disorder (SCI-IGD) tool was used to measure IGD. The SCI-IGD tool, consisting of 12 dichotomous questions, was developed by Koo, Han, Park, and Kwon (41) to diagnose IGD in accordance with DSM-5 diagnostic criteria and determine whether the 9-diagnostic criteria presented in DSM-5 are met. Cho et al. (42) verified the validity of the tool in adults. The diagnostic criteria of IGD in the DSM-5 comprises nine items: preoccupation, withdrawal, tolerance, unsuccessful attempts to control, loss of other interests, continued excessive use despite psychosocial problems, deception during online gaming, escape, and functional impairment (41). Participants who responded “yes” to five or more of the criteria were considered positive (IGD) and others were classified as negative (normal internet game users) during the last year in the nine-diagnostic criteria.

#### 2.2.2 Problematic game use

Problematic Game Use was assessed using the Game Overuse Screening Questionnaire (GOS-Q). The GOS-Q is a more comprehensive and definitive screening tool used to distinguish high-risk independent users associated with overuse of games (such as PC games, console games, smartphone games, internet/online games) from casual internet users. The GOS-Q comprises a 30-item questionnaire, and these 30 items are classified into six subscales (preoccupation, tolerance, impaired interpersonal domain, impairment in occupational or academic domain, loss of control, and loss of interest in other areas) (43). Respondents were asked to state the degree to which they had experienced symptoms in the past month through a 30 item-questionnaire (e.g., “I tried to reduce the amount of time I play games, but it is difficult”), using a four-point Likert scale (the four points were: not at all, sometimes, often, always) and with a total score ranging from 30 to 120 points. The Cronbach’s alpha of the GOS-Q was 0.96, and respondents who scored 38.5 points or more were considered high-risk indicators (43). This study classified participants who had not played any games within the last month or played games but scored less than 38.5 points in the GOS-Q as “negative” and those who scored 38.5 points or more as “positive”.

#### 2.2.3 Psychiatric disorders

Psychiatric disorders were measured using the Korean version of the Composite International Diagnostic Interview 2.1 (CIDI) (44). The CIDI (45) is a fully structured diagnostic tool that diagnoses psychiatric disorders based on the DSM-IV (46). The lifetime and one-year prevalence of alcohol use, nicotine use, depression, and anxiety disorders were calculated and analyzed.

#### 2.2.4 ADHD

We used the Adult ADHD Self-Report Scale-Version 1.1 (ASRS-v1.1) Screener (47) to screen adults with ADHD. The ASRS-v1.1 was developed by revising the WHO CIDI (48) for the WHO World Mental Health Survey Initiative (49). Each question in the ASRS-v1.1 Screener asks respondents to rate the frequency of a particular symptom of ADHD in the previous six months using a five-point Likert scale consisting of “never,” “rarely,” “sometimes,” “often,” and “very often.” The first three items were considered symptomatic if the respondents answered “sometimes,” “often,” or “very often,” and for the last three items, they were considered symptomatic if they answered “often” or “very often.” If respondents had symptoms for more than four of the six items, they were considered to have adult ADHD (50).

#### 2.2.5 Socio-demographic characteristics

Self-reported questionnaires from the NMHSK 2021 were used to obtain information on socio-demographic characteristics. The variables included gender (male/female), age (mean age), education (high school degree/lower/college degree/higher), employment (full-time/part-time/unemployed), and family income (below standard median income/above standard median income).

#### 2.2.6 Physical activity

This study used the Korean version of the short form of the International Physical Activity Questionnaire (IPAQ), which measures health-related physical activity in populations (51).The reliability of the Korean version of the IPAQ was confirmed by the Spearman Rho coefficients 0.427 to 0.646 (median value 0.542) and the Kappa value 0.365 to 0.620 (median value 0.471) (52). Using this measure, this study assessed high-intensity activities, moderated activities, and walking hours, which were conducted for more than 10 minutes in the last 7 days, and the corresponding average duration in hours. Respondents’ activity levels were classified into three groups (low/intermediate/high).

#### 2.2.7 Subjective and psychological characteristics

Quality of life was measured by asking, “How would you evaluate your quality of life?” Respondents answered “very bad,” “bad,” “not bad,” “good,” or “very good,” and classified the answer into “satisfaction” (not bad/good/very good) or “dissatisfaction” (very bad/bad).

Resilience was measured using the 10-item Connor-Davidson Resilience Scale (CD-RISC), which was extracted from the original 25-item CD-RISC (53). Reliability and validity of the 10-item Korean version of the CD-RISC were assessed, with the Cronbach’s alpha coefficient and test-retest reliability being 0.93 and 0.93, respectively (54). Each item was rated on a five-point Likert scale ranging from 0 (not true at all) to 4 (true nearly all the time). Total scores were obtained by summing all the responses and ranged from 0 to 40, with higher scores indicating greater resilience (55).

The Loneliness and Social Isolation Scale (LSIS) (56) was used to evaluate loneliness and social isolation. The scale consists of six self-reported items evaluated using three subscales: loneliness (two items), social support (two items), and social networks (two items) (56). Each item was rated on a four-point Likert scale ranging from 0 to 3. The greater the sum of each sub-factor, the more the loneliness, the less the social support, and the smaller the size of the social network perceived over the past month. Cronbach’s alpha for the LSIS was 0.774 (56).

### 2.3 Statistical analysis

A complex sample design was utilized for the statistical analyses. The Rao-Scott Chi-square test was performed to compare gender, educational status, employment status, family income, physical activity, and the quality of life among participants with IGD, problematic game users, and normal game users. A t-test was performed to compare the mean differences in age, resilience, and loneliness and social isolation among participants with IGD, problematic game users, and normal game users. Multivariate logistic regression analyses were performed to calculate the odds ratios (ORs) and 95% confidence intervals (CIs) of the 12-month prevalence of psychiatric disorders and the 6-month prevalence of ADHD based on the presence of IGD and problematic game use. Moreover, adjusted ORs were calculated after correcting for sociodemographic, physical activity, and psychological variables that appeared significantly different among the groups (i.e., IGD vs. normal game users and problematic game users vs. normal game users). We used SPSS (version 26, SPSS; IBM Co., Armonk, NY, USA) to perform the statistical analyses, and a p-value less than 0.05 was considered significant.

## 3 Results

### 3.1 Prevalence, correlates, and comorbidities of IGD

The estimated prevalence rate of IGD was 0.8% (22/2764). Participants with IGD were more likely male (p=0.003), had a lower mean age (p=0.001), were unemployed (p=0.018), had low physical activity (p<0.001), were dissatisfied with their quality of life (p=0.002), and perceived more loneliness and social isolation (p=0.026) compared to the normal game users (**Table 1**).

**Table 1:** Characteristics of subjects with and without IGD.

			IGD				
			Total (n=2,764) (n, weighted %)	Negative (n=2,742) (n, weighted %)	Positive (n=22) (n, weighted %)	Rao-Scott χ^2^/ t	p-value
Socio-demographic characteristics	Gender	Male	1,403 (51.6)	1,384 (51.3)	19 (85.7)	12.550	0.003
		Female	1,361 (48.4)	1,358 (48.7)	3 (14.3)		
	Age	Mean (S.E.)	34.64 (0.136)	34.70 (0.138)	28.23 (1.467)	3.435	0.001
	Education	High School degree or lower	977 (35.2)	968 (34.8)	9 (41.1)	0.411	0.591
		College degree or higher	1,787 (64.8)	1,774 (64.9)	13 (58.9)		
	Employment	Full-time	1,707 (61.2)	1,697 (61.4)	10 (37.8)	12.635	0.018
		Part-time	395 (13.3)	394 (13.3)	1 (6.8)		
		Unemployed	662 (25.5)	651 (25.2)	11 (55.3)		
	Family income	<Standard median income	1,069 (35.6)	1,061 (35.5)	8 (37.9)	0.063	0.834
		≥Standard median income	1,695 (64.4)	1,681 (64.5)	14 (62.1)		
Physical activity	IPAQ	Low	845 (29.6)	832 (29.2)	13 (69.4)	20.617	<0.001
		Intermediate	1,268 (47.8)	1,262 (48.0)	6 (23.4)		
		High	651 (22.6)	648 (22.8)	3 (7.2)		
Subjective & psychological characteristics	Quality of life	Dissatisfaction	850 (29.5)	837 (29.2)	13 (61.1)	12.970	0.002
		Satisfaction	1914 (70.5)	1905 (70.8)	9 (38.9)		
	Resilience	Mean (S.E.)	26.09 (0.222)	26.13 (0.223)	22.38 (1.474)	1.061	0.289
	LSIS	Mean (S.E.)	7.70 (0.092)	7.67 (0.091)	10.75 (1.236)	-2.231	0.026

IPAQ, International Physical Activity Questionnaire; LSIS, Loneliness and Social Isolation Scale.

AUD (OR 6.451, 95% CI 1.784–23.326), anxiety disorder (OR 3.533, 95% CI 1.038-12.022), and ADHD (OR 18.719, 95% CI 6.391–54.824) were significantly related to IGD. After adjusting for gender, age, employment, IPAQ, quality of life, and LSIS, AUD (OR 3.832, 95% CI 1.127–13.035) and ADHD (OR 12.320, 95% CI 2.583–58.757) were still significantly correlated with IGD, but there was no association between anxiety disorder and IGD (**Table 2**).

**Table 2:** Odds ratios for psychiatric disorders among subjects with and without IGD.

		IGD									
		Total (n=2,764) (n, weighted %)	Negative (n=2,742) (n, weighted %)	Positive (n=22) (n, weighted %)	Unadjusted			Adjusted			
					OR	95% CI	p-value	OR	95% CI	p-value	
Alcohol use disorder	Negative	2,678 (96.6)	2,659 (96.8)	19 (82.3)							
	Positive	86 (3.4)	83 (3.2)	3 (17.7)	6.451	1.784-23.326	0.005	3.832	1.127-13.035	0.032	
Nicotine use disorder	Negative	2,680 (96.7)	2,660 (96.8)	20 (88.6)							
	Positive	84 (3.3)	82 (3.2)	2 (11.4)	3.932	0.884-17.497	0.072	1.863	0.474-7.317	0.373	
Depressive disorder	Negative	2,722 (98.5)	2,702 (98.6)	20 (94.6)							
	Positive	42 (1.5)	40 (1.4)	2 (5.4)	3.926	0.640-24.081	0.139	1.431	0.239-8.551	0.694	
Anxiety disorder	Negative	2,662 (96.5)	2,644 (96.6)	18 (89.0)							
	Positive	102 (3.5)	98 (3.4)	4 (11.0)	3.533	1.038-12.022	0.043	3.617	0.972-13.451	0.055	
ADHD symptoms	Negative	2,702 (97.5)	2,686 (97.7)	16 (69.6)							
	Positive	62 (2.5)	56 (2.3)	6 (30.4)	18.719	6.391-54.824	<0.001	12.320	2.583-58.757	0.002	

Adjusted OR adjusted for gender, age, employment, IPAQ, quality of life, and LSIS.

### 3.2 Prevalence, correlates, and comorbidities of problematic game use

The estimated prevalence rate of problematic game use in the past month was 8.4% (232/2764). As shown in **Table 3**, the estimates for problematic game use were significantly higher among male participants (p<0.001), those who had a lower mean age (p<0.001), those who had a college degree or higher (p=0.002), the unemployed (p=0.001), those who had a higher family income (p=0.001), those with lower resilience (p=0.033), and those with perceived more loneliness and social isolation (p=0.037). AUD (OR 2.020, 95% CI 1.077-3.790) and ADHD (OR 3.943, 95% CI 2.226-6.983) were significantly correlated with problematic game use. After adjusting for gender, age, education, employment, family income, resilience, and LSIS, ADHD was still significantly correlated with problematic game use (OR 5.546, 95% CI 2.828-10.876), but there was no association between AUD and problematic game use disappeared (**Table 4**).

**Table 3:** Characteristics of subjects with and without problematic game use.

			Problematic Game Use				
			Total (n=2,764) (n, weighted %)	Negative (n=2,532) (n, weighted %)	Positive (n=232) (n, weighted %)	Rao-Scott χ^2^/ t	p-value
Socio-demographic characteristics	Gender	Male	1,403 (51.6)	1,224 (49.0)	179 (74.7)	66.796	<0.001
		Female	1,361 (48.4)	1,308 (51.0)	53 (25.3)		
	Age	Mean (S.E.)	34.64 (0.136)	35.35 (0.155)	28.38 (0.550)	9.840	<0.001
	Education	High School degree or lower	977 (35.2)	875 (34.0)	102 (45.7)	15.267	0.002
		College degree or higher	1,787 (64.8)	1,657 (66.0)	130 (54.3)		
	Employment	Full-time	1,707 (61.2)	1,568 (61.8)	139 (55.7)	20.768	0.001
		Part-time	395 (13.3)	372 (13.8)	23 (8.3)		
		Unemployed	662 (25.5)	592 (24.3)	70 (36.0)		
	Family income	<Standard median income	1,069 (35.6)	1,000 (36.7)	69 (25.9)	12.883	0.001
		≥Standard median income	1,695 (64.4)	1,532 (63.3)	163 (74.1)		
Physical activity	IPAQ	Low	845 (29.6)	755 (28.8)	90 (36.7)	7.761	0.078
		Intermediate	1,268 (47.8)	1,171 (48.2)	97 (43.9)		
		High	651 (22.6)	606 (23.0)	45 (19.5)		
Subjective & psychological characteristics	Quality of life	Dissatisfaction	850 (29.5)	778 (29.7)	72 (27.8)	0.440	0.606
		Satisfaction	1,914 (70.5)	1,754 (70.3)	160 (72.2)		
	Resilience	Mean (S.E.)	26.09 (0.222)	26.26 (0.227)	24.57 (0.570)	2.131	0.033
	LSIS	Mean (S.E.)	7.70 (0.092)	7.66 (0.095)	8.08 (0.256)	-2.094	0.037

IPAQ, International Physical Activity Questionnaire; LSIS, Loneliness and Social Isolation Scale.

**Table 4:** Odds ratios for psychiatric disorders among subjects with and without problematic game use.

		Problematic Game Use								
		Total (n=2,764) (n, weighted %)	Negative(n=2,532) (n, weighted %)	Positive(n=232) (n, weighted %)	Unadjusted			Adjusted		
					OR	95% CI	p-value	OR	95% CI	p-value
Alcohol use disorder	Negative	2,678(96.6)	2,461(96.9)	217(94.0)						
	Positive	86(3.4)	71(3.1)	15(6.0)	2.020	1.077-3.790	0.029	1.358	0.684-2.699	0.381
Nicotine use disorder	Negative	2,680(96.7)	2,458(96.9)	220(95.5)						
	Positive	84(3.3)	74(3.1)	10(4.5)	1.450	0.685-3.067	0.331	0.962	0.417-2.221	0.928
Depressive disorder	Negative	2,722(98.5)	2,497(98.6)	225(98.2)						
	Positive	42(1.5)	35(1.4)	7(1.8)	1.259	0.498-3.184	0.626	1.347	0.398-4.562	0.631
Anxiety disorder	Negative	2,662(96.5)	2,443(96.7)	219(95.3)						
	Positive	102(3.5)	89(3.3)	13(4.7)	1.425	0.720-2.822	0.309	1.641	0.740-3.642	0.223
ADHD symptoms	Negative	2,702(97.5)	2,487(98.0)	215(92.6)						
	Positive	62(2.5)	45(2.0)	17(7.4)	3.943	2.226-6.983	<0.001	5.546	2.828-10.876	<0.001

Adjusted OR adjusted for gender, age, education, employment, family income, resilience, and LSIS.

## 4 Discussion

This study investigated the prevalence, correlates, and comorbidities of a wide range of psychiatric disorders of IGD and problematic game use in a nationally representative sample of Korean adults. We extended the findings of previous studies on IGD and problematic game use by using a nationwide sample of young adults and by applying the DSM-5 criteria for IGD and the GOS-Q.

The prevalence rate of IGD was 0.8% in our finding, which is like the rates reported in the United States and European countries (10). According to data from large international adult cohorts, the 6-month prevalence rates of IGD were estimated to be between 0.32% and 1.04% (10). On the other hand, the prevalence rate of 13.8% yielded by a previous Korean study (8) is much higher when compared to the rate from the current study. This variation may be due to the different sampling populations used in the two studies. While our data were from a nationally representative sample that included the general population of Korean adults, Kim et al. (8) employed data from an online survey with inclusion criteria specifically targeting internet gamers in fact, a previous systematic review study has shown that the prevalence rate of IGD is higher among a sample of only gamers compared to a mixed sample of gamers and non-gamers (14). Additionally, the survey methods may also account for the differences in prevalence rates across studies (14). Online surveys tend to yield higher prevalence compared to other methods such as classroom, mail, or telephone surveys. Since the data used in our study was collected through face-to-face interviews as part of the NMHSK 2021, the prevalence rate in our study may be lower than those reported by Kim et al. (8).

Meanwhile, the prevalence rate of problematic game use was 8% in the current study, which is almost twice as low as the 2016 prevalence of 17.6% using the same measurement and cut-off point (38). This discrepancy in prevalence rates may be due to the different number of subjects (N=415) and age range (18–30 years old) in the previous research, as well as the gaming regulatory policies in Korea. In 2014, regulations were implemented for in-game purchases, particularly for online board games, to reduce overconsumption and prevent games from being used as a means of gambling (57). Given that the in-game purchases showed a significantly positive association with probable gaming disorder (58), it is plausible that consistent restriction of in-game purchases may have contributed to the decrease in problematic game use prevalence.

Compared to normal use, IGD and problematic game use were more prevalent in males than in females (14, 17, 18). Darvesh et al. (9) reviewed 160 studies that used 35 different methods to diagnose IGD; these studies, conducted in general, clinical, and severe populations, highlighted prevalence rates of 0.21–57.5% for males in the general population who were not undergoing treatment for IGD. Most studies have described a higher prevalence of IGD and problematic game use among young people than among the elderly, with an “age at risk” (14–16). Consistent with prior findings, participants with IGD were more likely to be unemployed (59), have lower physical activity, and be dissatisfied with their quality of life (14, 22). Loneliness and social isolation were found to be higher with both IGD and problematic game use than in normal use (60–62); this phenomenon was particularly noticeable with IGD. In addition, problematic game users showed lower resilience than normal game users. This may be because socially isolated and less resilient people cope by indulging in games rather than social activities (63–65). Further, participants with problematic game use had higher education levels. These findings were inconsistent with those from earlier studies (39, 66). Another study found game addiction to be independent of educational background (21, 67) and there were no significant differences (38). However, a medium or high education level showed a significant positive association with excessive computer game playing among Norwegian adults (68). Meanwhile, in this study, a group with high family income showed an association with problematic game use. In a systematic literature review study by Kuss et al. (69), high household income in the youth population group was related to internet addiction, and in a study by Ju et al. (70), the internet use index was significantly related to income level. The lower the income group, the lower the probability of accessing the internet at home, and the higher the probability of access at high income. Therefore, there is a gap in internet use according to income level. There is a paucity of up-to-date studies on problematic game use and socio-economic status based on young adults. More research is needed to identify the sociodemographic factors relevant to the risk of developing gaming disorder through longitudinal data.

Previous studies (4, 30, 71, 72) stated that IGD was associated with an increased prevalence of AUD. This association between IGD and AUD may be attributed to several shared psychological features, including dysfunctional impulsivity, limited capacity for self-control, excessive anxiety, and severe psychopathological impairment (30, 73). Evidence supports the co-occurrence of addiction for both substances and behaviors, in particular, the presence of a behavioral addiction increases the propensity for addiction to develop other behaviors (74). This may create a cycle of reciprocity, wherein mutual exacerbation occurs between two or more problematic behaviors (75, 76). Na et al. (30) surveyed South Korean adults (n=1,819) online and found that 21% experienced both problematic alcohol use and problematic game use. Our results revealed a significant association between AUD and IGD, a more severe condition than problematic game use.

The findings suggested that ADHD is the most frequent psychiatric comorbidity of IGD and problematic game use (5, 34–36). Recent cross-sectional studies with community samples found that ADHD symptoms were closely associated with IGD symptoms (77–80). Many studies have reported psychiatric comorbidities and a conceptual overlap between gaming disorder and ADHD in terms of clinical and biological aspects (34, 35, 81, 82). ADHD features such as impulsivity, low self-esteem, hyperactivity, and inattentiveness can contribute to the development of IGD (83). Several community-based longitudinal studies indicated that ADHD was a predictor of the development of IGD and problematic game use (84–86). Lee et al. (5) showed that ADHD comorbidity was associated with a more chronic course of IGD, with lower rates of recovery and higher rates of recurrence. In a longitudinal clinical cohort study, Han et al. (87) investigated 755 patients with IGD over five years and found that the comorbid condition of ADHD was one of the key predictive factors for long-term recovery from IGD. In a three-year clinical cohort study, ADHD comorbidity predicted a poor clinical course of IGD, wherein changes in ADHD symptoms were associated with alterations in IGD symptoms (33). ADHD and IGD share similar biological features (81, 82, 88). Several resting-state functional magnetic resonance imaging (MRI) studies on the shared conditions between ADHD and IGD illustrated diminished functional connectivity within the attentional network and cortico-subcortical circuits (81, 82). Furthermore, Han et al. (88) conducted a one-year longitudinal study of IGD and ADHD subjects and reported similar changes in functional connectivity between the cortex and subcortex in response to treatment. Due to high comorbidity and the negative impact of ADHD symptoms on the course of IGD, patients with IGD and problematic game use require clinical attention as a high-risk group as well as extensive management for their mental difficulties.

The strengths of this study are as follows: This study is the first in Korea to measure the prevalence, correlation, and comorbidities by using DSM-5 internet game disorder diagnostic criteria and a tool to screen for game addiction among the adult general population via nationally representative data. Moreover, the data used in this study, well-trained interviewers collected through face-to-face interview with participants. The limitations of this study were as follows. First, due to its cross-sectional design, this study could not determine causality among variables. For instance, our data could not explain how psychiatric comorbidities contributed to IGD and problematic game use or, conversely, how addictive symptoms affected the progression of other psychiatric symptoms. Second, our data focused on the Korean adult population, thereby excluding the adolescent population. Given that IGD and problematic game use are more prevalent and severe among adolescents (89), our results may not fully reflect the magnitude of the gaming addiction problem in Korea. Last, our results, obtained from a Korean sample, may have been influenced by social and cultural environment characteristics.

## 5 Conclusion

Our results revealed that IGD and problematic game use are relatively prevalent in the Korean adult population and are highly comorbid with AUD and ADHD. Therefore, a preventive strategy for IGD and problematic game use is needed for game users who are more likely to be addicted, such as being younger and male. In addition, mental health screening and appropriate treatment for both game addiction and comorbid psychiatric disorder are required for individuals with IGD and problematic game users.

## Data Availability

The data analyzed in this study is subject to the following licenses/restrictions: Raw data were generated at National Center for Mental Health. Derived data supporting the findings of this study are available from the corresponding author SP on request.

## References

[1] van den Brink W. ICD-11 Gaming Disorder: Needed and just in time or dangerous and much too early? Commentary on: Scholars’ open debate paper on the World Health Organization ICD-11 Gaming Disorder proposal (Aarseth et al.). J Behav Addict. (2017) 6:290–2. doi: 10.1556/2006.6.2017.040 PMC5700734 28033714

[2] American Psychiatric Association. Diagnostic and Statistical Manual. 5th ed. Washington, DC: American Psychiatric Publishing (2013). doi: 10.1176/app.books.9780890425596

[3] World Health Organization. ICD-11, the 11th Revision of the International Classification of Diseases (2018). Available online at: https://icd.who.int/ (Accessed April 6, 2024).

[4] Müller KW Wölfling K. Both sides of the story: Addiction is not a pastime activity: Commentary on: Scholars’ open debate paper on the World Health Organization ICD-11 Gaming Disorder proposal (Aarseth et al.). J Behav Addict. (2017) 6:118–20. doi: 10.1556/2006.6.2017.038 PMC5520131 28662617

[5] Lee J Bae S Kim BN Han DH. Impact of attention-deficit/hyperactivity disorder comorbidity on longitudinal course in Internet gaming disorder: a 3-year clinical cohort study. J Child Psychol Psychiatry. (2021) 62:1110–9. doi: 10.1111/jcpp.13380 33751554

[6] Rehbein F Kliem S Baier D Mößle T Petry NM. Prevalence of internet gaming disorder in German adolescents: Diagnostic contribution of the nine DSM-5 criteria in a state-wide representative sample. Addict. (2015) 110:842–51. doi: 10.1111/add.12849 25598040

[7] Lemmens JS Valkenburg PM Gentile DA. The internet gaming disorder scale. Psychol Assess. (2015) 27:567–82. doi: 10.1037/pas0000062 25558970

[8] Kim NR Hwang SSH Choi JS Kim DJ Demetrovics Z Király O. Characteristics and psychiatric symptoms of internet gaming disorder among adults using self-reported DSM-5 criteria. Psychiatry Investig. (2016) 13:58–66. doi: 10.4306/pi.2016.13.1.58 PMC4701686 26766947

[9] Darvesh N Radhakrishnan A Lachance CC Nincic V Sharpe JP Ghassemi M. Exploring the prevalence of gaming disorder and Internet gaming disorder: A rapid scoping review. Syst Rev. (2020) 9:68. doi: 10.1186/s13643-020-01329-2 32241295 PMC7119162

[10] Przybylski AK Weinstein N Murayama K. Internet gaming disorder: Investigating the clinical relevance of a new phenomenon. Am J Psychiatry. (2017) 174:230–6. doi: 10.1176/appi.ajp.2016.16020224 27809571

[11] Lee D Hong SJ Jung YC Park J Kim IY Namkoong K. Altered heart rate variability during gaming in internet gaming disorder. Cyberpsychol Behav Soc Netw. (2018) 21:259–67. doi: 10.1089/cyber.2017.0486 29624440

[12] Griffiths MD van Rooij AJ Kardefelt-Winther D Starcevic V Király O Pallesen S. Working towards an international consensus on criteria for assessing internet gaming disorder: A critical commentary on Petry et al. Addict. (2016) 111:167–75. doi: 10.1111/add.13057 PMC5699464 26669530

[13] Festl R Scharkow M Quandt T. Problematic computer game use among adolescents, younger and older adults. Addict. (2013) 108:592–9. doi: 10.1111/add.12016 23078146

[14] Mihara S Higuchi S. Cross-sectional and longitudinal epidemiological studies of Internet gaming disorder: A systematic literature review. Psychiatry Clin Neurosci. (2017) 71:425–44. doi: 10.1111/pcn.12532 28436212

[15] González-Bueso V Santamaría JJ Oliveras I Fernández D Montero E Baño M. Internet gaming disorder clustering based on personality traits in adolescents and its relation with comorbid psychological symptoms. Int J Environ Res Public Health. (2020) 17:1516. doi: 10.3390/ijerph17051516 32111070 PMC7084409

[16] van den Eijnden R Koning I Doornwaard S van Gurp F ter Bogt T. The impact of heavy and disordered use of games and social media on adolescents’ psychological, social, and school functioning. J Behav Addict. (2018) 7:697–706. doi: 10.1556/2006.7.2018.65 30264607 PMC6426368

[17] Bouna-Pyrrou P Aufleger B Braun S Gattnar M Kallmayer S Wagner H. Cross-sectional and longitudinal evaluation of the social network use disorder and internet gaming disorder criteria. Front Psychiatry. (2018) 9:692. doi: 10.3389/fpsyt.2018.00692 30627106 PMC6310011

[18] Dong G-H Potenza MN. Considering gender differences in the study and treatment of internet gaming disorder. J Psychiatr Res. (2022) 153:25–9. doi: 10.1016/j.jpsychires.2022.06.057 35793576

[19] Marraudino M Bonaldo B Vitiello B Bergui GC Panzica G. Sexual differences in internet gaming disorder (IGD): From psychological features to neuroanatomical networks. J Clin Med. (2022) 11:1018. doi: 10.3390/jcm11041018 35207293 PMC8877403

[20] Rho MJ Lee H Lee T-H Cho H Jung DJ Kim D-J. Risk factors for internet gaming disorder: psychological factors and internet gaming characteristics. Int J Environ Res Public Health. (2018) 15:40. doi: 10.3390/ijerph15010040 PMC5800139 29280953

[21] Wack E Tantleff-Dunn S. Relationships between electronic game play, obesity, and psychosocial functioning in young men. Cyberpsychol Behav. (2009) 12:241–4. doi: 10.1089/cpb.2008.0151 19006465

[22] Mentzoni RA Brunborg GS Molde H Myrseth H Skouverøe KJM Hetland J. Problematic video game use: Estimated prevalence and mental and physical health associations. Cyberpsychol Behav Soc Netw. (2011) 14:591–6. doi: 10.1089/cyber.2010.0260 21342010

[23] Kuss DJ Griffiths MD. Internet and gaming addiction: A systematic literature review of neuroimaging studies. Brain Sci. (2012) 2:347–74. doi: 10.3390/brainsci2030347 PMC4061797 24961198

[24] Kuss DJ Pontes HM Griffiths MD. Neurobiological correlates in internet gaming disorder: A systematic literature review. Front Psychiatry. (2018) 9:166. doi: 10.3389/fpsyt.2018.00166 29867599 PMC5952034

[25] Rehbein F Staudt A Hanslmaier M Kliem S. Video game playing in the general adult population of Germany: Can gender specific genre preferences explain higher gaming time of males? Comput Hum Behav. (2016) 55:729–35. doi: 10.1016/j.chb.2015.10.016

[26] Green KT Calhoun PS Dennis MF Beckham JC Miller-Mumford M Fernandez A. Exploration of the resilience construct in posttraumatic stress disorder severity and functional correlates in military combat veterans who have served since September 11, 2001. J Clin Psychiatry. (2010) 71:823–30. doi: 10.4088/JCP.09m05780blu 20584523

[27] Wingo AP Ressler KJ Bradley B. Resilience characteristics mitigate the tendency for harmful alcohol and illicit drug use in adults with a history of childhood abuse: a cross-sectional study of 2024 inner-city men and women. J Psychiatry Res. (2014) 51:93–9. doi: 10.1016/j.jpsychires.2014.01.007 PMC4605671 24485848

[28] King DL Haagsma MC Delfabbro PH Gradisar M Griffiths MD. Toward a consensus definition of pathological video-gaming: a systematic review of psychometric assessment tools. Clin Psychol Rev. (2013) 33:331–42. doi: 10.1016/j.cpr.2013.01.002 23396015

[29] Adams BL Stavropoulos V Burleigh TL Liew LW Beard CL Griffiths MD. Internet gaming disorder behaviors in emergent adulthood: A pilot study examining the interplay between anxiety and family cohesion. Int J Ment Health Addict. (2019) 17:828–44. doi: 10.1007/s11469-018-9873-0

[30] Na E Lee H Choi I Kim D-J. Comorbidity of Internet gaming disorder and alcohol use disorder: a focus on clinical characteristics and gaming patterns. Am J Addict. (2017) 26:326–34. doi: 10.1111/ajad.12528 28328110

[31] Schimmenti A Infanti A Badoud D Laloyaux J Billieux J. Schizotypal personality traits and problematic use of massively multiplayer online role-playing games (MMORPGs). Comput Hum Behav. (2017) 74:286–93. doi: 10.1016/j.chb.2017.04.048

[32] Porter G Starcevic V Berle D Fenech P. Recognizing problem video game use. Aust N Z J Psychiatry. (2010) 44:120–8. doi: 10.3109/00048670903279812 20113300

[33] Fitz-Walter Z Johnson D Wyeth P Tjondronegoro D Scott-Parker B. Driven to drive? Investigating the effect of gamification on learner driver behavior, perceived motivation and user experience. Comput Hum Behav. (2017) 71:586–95. doi: 10.1016/j.chb.2016.08.050

[34] Dullur P Krishnan V Diaz AM. A systematic review on the intersection of attention-deficit hyperactivity disorder and gaming disorder. J Psychiatr Res. (2021) 133:212–22. doi: 10.1016/j.jpsychires.2020.12.026 33360866

[35] Starcevic V Khazaal Y. Editorial: problematic Gaming, personality, and psychiatric disorders. Front Psychiatry. (2020) 10:1004. doi: 10.3389/fpsyt.2019.01004 32010002 PMC6979487

[36] Yen J-Y Liu T-L Wang P-W Chen C-S Yen C-F Ko C-H. Association between internet gaming disorder and adult attention deficit and hyperactivity disorder and their correlates: impulsivity and hostility. Addict Behav. (2017) 64:308–13. doi: 10.1016/j.addbeh.2016.04.024 27179391

[37] Rim SJ Hahm BJ Seong SJ Park JE Chang SM Kim BS. Prevalence of mental disorders and associated factors in Korean adults: National mental health survey of Korea 2021. Psychiatry Investig. (2023) 20:262–72. doi: 10.30773/pi.2022.0307 PMC10064208 36990670

[38] Byeon G Park JE Jeon HJ Seong SJ Lee DW Cho SJ. Associations between game use and mental health in early adulthood: A nationwide study in Korea. J Affect Disord. (2022) 297:579–85. doi: 10.1016/j.jad.2021.10.064 34737016

[39] Wittek CT Finserås TR Pallesen S Mentzoni RA Hanss D Griffiths MD. Prevalence and predictors of video game addiction: A study based on a national representative sample of gamers. Int J Ment Health Addict. (2016) 14:672–86. doi: 10.1007/s11469-015-9592-8 PMC5023737 27688739

[40] Jeong YW Han YR Kim SK Jeong HS. The frequency of impairments in everyday activities due to the overuse of the internet, gaming, or smartphone, and its relationship to health-related quality of life in Korea. BMC Public Health. (2020) 20:1–16. doi: 10.1186/s12889-020-08922-z 32552690 PMC7301989

[41] Koo HJ Han DH Park S-Y Kwon J-H. The structured clinical interview for DSM-5 internet gaming disorder: Development and Validation for diagnosing IGD in adolescents. Psychiatry Investig. (2017) 14:21–9. doi: 10.4306/pi.2017.14.1.21 PMC5240456 28096871

[42] Cho SH Kwon J-H. The validation of structured clinical interview for internet gaming disorder (SCI-IGD) and evaluation of the DSM-5 internet gaming disorder criteria: Findings from a community sample of adults. Kor J Clin Psychol. (2016) 35:831–42. doi: 10.15842/kjcp.2016.35.4.011

[43] Baek I-C Kim J-H Joung Y-S Lee H-W Park S Park EJ. Development and validation study of game overuse screening questionnaire. Psychiatry Res. (2020) 290:113165. doi: 10.1016/j.psychres.2020.113165 32559564

[44] Cho MJ Hahm BJ Suh DW Hong JP Bae JN Kim JK. Development of the korean version of the composite international diagnostic interview (K-CIDI). J Korean Neuropsychiatr Assoc. (2002) 41:123–37.

[45] World Health Organization. Composite international Diagnostic Interview (CIDI): Version 1.0. Geneva, CH: World Health Organization (1990).

[46] American Psychiatric Association. Diagnostic and statistical manual of mental disorders (DSM-IV). Washington, DC: American Psychiatric Publishing (1994).

[47] Kessler RC Adler LA Barkley R Biederman J Conners CK Faraone SV. Patterns and predictors of attention-deficit/hyperactivity disorder persistence into adulthood: Results from the national comorbidity survey replication. Biol Psychiatry. (2005) 57:1442–51. doi: 10.1016/j.biopsych.2005.04.001 PMC2847347 15950019

[48] Kessler RC Üstün TB. The World Mental Health (WMH) survey initiative version of the World Health Organization (WHO) Composite International Diagnostic Interview (CIDI). Int J Methods Psychiatr Res. (2004) 13:93–121. doi: 10.1002/mpr.168 15297906 PMC6878592

[49] Alonso J Angermeyer MC Bernert S Bruffaerts R Brugha TS Bryson H. Prevalence of mental disorders in Europe: Results from the European Study of the Epidemiology of Mental Disorders (ESEMeD) project. Acta Psychiatr Scand. (2004) 109:21–7. doi: 10.1111/j.1600-0047.2004.00327.x 15128384

[50] Kessler RC Adler L Barkley R Biederman J Conners CK Demler O. The prevalence and correlates of adult ADHD in the United States: Results from the national comorbidity survey replication. Am J Psychiatry. (2006) 163:716–23. doi: 10.1176/ajp.2006.163.4.716 PMC2859678 16585449

[51] Hagströmer M Oja P Sjöström M. The International Physical Activity Questionnaire (IPAQ): A concurrent and construct validity study. Public Health Nutr. (2006) 9:755–62. doi: 10.1079/phn2005898 16925881

[52] Oh JY Yang YJ Kim BS Kang JH. Validity and reliability of the Korean version of the International Physical Activity Questionnaire (IPAQ) short form. J Korean Acad Fam Med. (2007) 28:532–41.

[53] Campbell-Sills L Stein MB. Psychometric analysis and refinement of the Connor–Davidson Resilience Scale (CD-RISC): Validation of a 10-item measure of resilience. J Trauma Stress. (2007) 20:1019–28. doi: 10.1002/jts.20271 18157881

[54] Baek H-S Lee K-U Joo E-J Lee M-Y Choi K-S. Reliability and validity of the Korean version of the Connor-Davidson Resilience Scale. Psychiatry Investig. (2010) 7:109–15. doi: 10.4306/pi.2010.7.2.109 PMC2890864 20577619

[55] Wang L Shi Z Zhang Y Zhang Z. Psychometric properties of the 10-item Connor-Davidson Resilience Scale in Chinese earthquake victims. Psychiatry Clin Neurosci. (2010) 64:499–504. doi: 10.1111/j.1440-1819.2010.02130.x 20923429

[56] Hwang SJ Hong JP An JH Kim MH Jeong SH Chang H. Development and validation of loneliness and social isolation scale. J Korean Neuropsychiatr Assoc. (2021) 60:291–7. doi: 10.4306/jknpa.2021.60.4.291

[57] Hwang SG. The present state and improvement direction of the major regulations on internet game. J Media Law Ethics Policy Res. (2018) 17:1–35. doi: 10.26542/JML.2018.12.17.3.1

[58] Oka T Kubo T Murakami M Kobayashi N. The relationship of game genres, in-game purchases, and playing duration with probable gaming disorder in two independent, large-scale online surveys of Japanese adults. J Behav Addict. (2024) 13:205–14. doi: 10.1556/2006.2023.00076 PMC10988416 38197896

[59] Park S Jeon HJ Bae JN Seong SJ Hong JP. Prevalence and psychiatric comorbidities of internet addiction in a nationwide sample of Korean adults. Psychiatry Investig. (2017) 14:879–82. doi: 10.4306/pi.2017.14.6.879 PMC5714733 29209395

[60] Wang J-L Sheng J-R Wang H-Z. The association between mobile game addiction and depression, social anxiety, and loneliness. Front Public Health. (2019) 7:247. doi: 10.3389/fpubh.2019.00247 31552213 PMC6743417

[61] Baysak E Yertutanol FDK Dalğar İ Candansayar S. How game addiction rates and related psychosocial risk factors change within 2-years: A follow-up study. Psychiatry Investig. (2018) 15:984–90. doi: 10.30773/pi.2018.08.16 PMC6212699 30301305

[62] Hussain Z Griffiths MD Baguley T. Online gaming addiction: classification, prediction and associated risk factors. Addict Res Theory. (2012) 20:359–71. doi: 10.3109/16066359.2011.640442

[63] Cudo A Wojtasiński M Tużnik P Fudali-Czyż A Griffiths MD. The relationship between depressive symptoms, loneliness, self-control, and gaming disorder among Polish male and female gamers: The indirect effects of gaming motives. Int J Environ Res Public Health. (2022) 19:10438. doi: 10.3390/ijerph191610438 36012072 PMC9408588

[64] Çutuk S Sertbaş K Çutuk ZA. I am investigating the relationship between self-confidence, psychological resilience, and problematic internet use. Int J Soc Sci Educ Stud. (2020) 7:48–58. doi: 10.23918/ijsses.v7i4p48

[65] Canale N Marino C Griffiths MD Scacchi L Monaci MG Vieno A. The association between problematic online gaming and perceived stress: The moderating effect of psychological resilience. J Behav Addict. (2019) 8:174–80. doi: 10.1556/2006.8.2019.01 PMC7044594 30739461

[66] Ünübol H Koç AŞ Sayar GH Stavropoulos V Kircaburun K Griffiths MD. Measurement, profiles, prevalence, and psychological risk factors of problematic gaming among the Turkish community: A large-scale national study. Int J Ment Health Addict. (2021) 19:1662–82. doi: 10.1556/2006.2023.00076

[67] Rehbein F Kleimann M Mößle T. Prevalence and risk factors of video game dependency in adolescence: results of a German nationwide survey. Cyberpsychol Behav Soc Netw. (2010) 13:269–77. doi: 10.1089/cyber.2009.0227 20557246

[68] Wenzel HG Bakken IJ. Excessive computer game playing among Norwegian adults: Self-reported consequences of playing and association with mental health problems. Psychol Rep. (2009) 105:1237–47. doi: 10.2466/PR0.105.F.1237-1247 20229923

[69] Kuss JD Griffiths MD Karila L Billieux J. Internet addiction: A systematic review of epidemiological research for the last decade. Curr Pharm Des. (2014) 20:4026–52. doi: 10.2174/13816128113199990617 24001297

[70] Ju YW Kim YJ Cho CH. Digital divide in internet access and internet usage in Korea. JKAIS. (2011) 12:5601–13. doi: 10.5762/KAIS.2011.12.12.5601

[71] Škařupová K Blinka L Ťápal A. Gaming under the influence: An exploratory study. J Behav Addict. (2018) 7:493–8. doi: 10.1556/2006.7.2018.27 PMC6174600 29788755

[72] Erevik EK Torsheim T Andreassen CS Krossbakken E Vedaa Ø Pallesen S. The associations between low-level gaming, high-level gaming, and problematic alcohol use. Addict Behav Rep. (2019) 10:100186. doi: 10.1016/j.abrep.2019.100186 31193377 PMC6527943

[73] Choi S-W Kim HS Kim G-Y Jeon Y Park SM Lee J-Y. Similarities and differences among Internet gaming disorder, gambling disorder, and alcohol use disorder: A focus on impulsivity and compulsivity. J Behav Addict. (2014) 3:246–53. doi: 10.1556/jba.3.2014.4.6 PMC4291830 25592310

[74] Burleigh TL Griffiths MD Sumich A Stavropoulos V Kuss DJ. A systematic review of the co-occurrence of gaming disorder and other potentially addictive behaviors. Curr Addict Rep. (2019) 6:383–401. doi: 10.1007/s40429-019-00279-7

[75] Haylett SA Stephenson GM Lefever RMH. A study of addictive orientations using the shorter-PROMID questionnaire. Addict Behav. (2004) 29:61–71. doi: 10.1016/S0306-4603(03)00083-2 14667421

[76] Martin RJ Usdan S Cremeens J Vail-Smith K. Disordered gambling and co-morbidity of psychiatric disorders among college students: An examination of problem drinking, anxiety and depression. J Gambl Stud. (2014) 30:321–33. doi: 10.1007/s10899-013-9367-8 23430449

[77] Aggarwal S Saluja S Gambhir V Gupta S Satia SPS. Predicting the likelihood of psychological disorders in Player Unknown’s Battlegrounds (PUBG) players from Asian countries using supervised machine learning. Addict Behav. (2020) 101:106132. doi: 10.1016/j.addbeh.2019.106132 31704370

[78] Evren C Evren B Dalbudak E Topcu M Kutlu N. Relationships of internet addiction and internet gaming disorder symptom severities with probable attention-deficit/hyperactivity disorder, aggression and negative affect among university students. Atten Deficit Hyperact Disorder. (2019) 11:413–21. doi: 10.1007/s12402-019-00305-8 31062235

[79] Jung D Shim E-J Park H Lee K Lee S Kim E-Y. The association between excessive internet gaming behavior and immersive tendency, mediated by attention-deficit/hyperactivity disorder symptoms, in Korean male university students. Psychiatry Investig. (2020) 17:403–11. doi: 10.30773/pi.2019.0173 PMC7265032 32295328

[80] Stavropoulos V Adams BLM Beard CL Dumble E Trawley S Gomez R. Associations between attention deficit hyperactivity and internet gaming disorder symptoms: Is there consistency across types of symptoms, gender, and countries? Addict Behav Rep. (2019) 9:100158. doi: 10.1016/j.abrep.2018.100158 30671530 PMC6327637

[81] Castellanos FX Aoki Y. Intrinsic functional connectivity in Attention-Deficit/Hyperactivity Disorder: A science in development. Biol Psychiatry Cognit Neurosci Neuroimaging. (2016) 1:253–61. doi: 10.1016/j.bpsc.2016.03.004 PMC5047296 27713929

[82] Weinstein AM. An update overview on brain imaging studies of internet gaming disorder. Front Psychiatry. (2017) 8:185. doi: 10.3389/fpsyt.2017.00185 29033857 PMC5626837

[83] Michael T Seymour P. A brief comprehensive review of the interactions between Attention−Deficit/Hyperactivity disorder and internet gaming disorder. Int J Ment Health Addict. (2023). doi: 10.1007/s11469-023-01215-7

[84] Ferguson CJ Ceranoglu TA. Attention problems and pathological gaming: Resolving the ‘chicken and egg’ in a prospective analysis. Psychiatr Q. (2014) 85:103–10. doi: 10.1007/s11126-013-9276-0 24132870

[85] Peeters M Koning I van den Eijnden R. Predicting internet gaming disorder symptoms in young adolescents: A one-year follow-up study. Comp Hum Behav. (2018) 80:255–61. doi: 10.1016/j.chb.2017.11.008

[86] Wartberg L Kriston L Zieglmeier M Lincoln T Kammerl R. A longitudinal study on psychosocial causes and consequences of internet gaming disorder in adolescence. Psychol Med. (2018) 49:287–94. doi: 10.1017/S003329171800082X 29622057

[87] Han DH Yoo M Renshaw PF Petry NM. A cohort study of patients seeking internet gaming disorder treatment. J Behav Addict. (2018) 7:930–8. doi: 10.1556/2006.7.2018.102 PMC6376391 30418074

[88] Han DH Bae S Hong J Kim SM Son YD Renshaw P. Resting-state fMRI study of ADHD and internet gaming disorder. J Atten Disord. (2021) 25:1080–95. doi: 10.1177/1087054719883022 31640464

[89] Haagsma MC Pieterse ME Peters O. The prevalence of problematic video gamers in the Netherlands. Cyberpsychol Behav Soc Netw. (2012) 15:162–8. doi: 10.1089/cyber.2011.0248 22313358

